# Two stage treatment of dairy effluent using immobilized *Chlorella pyrenoidosa*

**DOI:** 10.1186/2052-336X-11-36

**Published:** 2013-12-19

**Authors:** Rajasri Yadavalli, Goutham Rao Venkata Naga Heggers

**Affiliations:** 1 Department of Biotechnology, Sreenidhi Institute of Science and Technology, Hyderabad, Andhra Pradesh 501301, India

**Keywords:** Dairy effluent, *Chlorella pyrenoidosa*, Immobilization, Photobioreactor, Sand bed filtration, Nutrient removal, Aquatic life, Biofertilizer

## Abstract

**Background:**

Dairy effluents contains high organic load and unscrupulous discharge of these effluents into aquatic bodies is a matter of serious concern besides deteriorating their water quality. Whilst physico-chemical treatment is the common mode of treatment, immobilized microalgae can be potentially employed to treat high organic content which offer numerous benefits along with waste water treatment.

**Methods:**

A novel low cost two stage treatment was employed for the complete treatment of dairy effluent. The first stage consists of treating the diary effluent in a photobioreactor (1 L) using immobilized *Chlorella pyrenoidosa* while the second stage involves a two column sand bed filtration technique.

**Results:**

Whilst NH_4_^+^-N was completely removed, a 98% removal of PO_4_^3-^-P was achieved within 96 h of two stage purification processes. The filtrate was tested for toxicity and no mortality was observed in the zebra fish which was used as a model at the end of 96 h bioassay. Moreover, a significant decrease in biological oxygen demand and chemical oxygen demand was achieved by this novel method. Also the biomass separated was tested as a biofertilizer to the rice seeds and a 30% increase in terms of length of root and shoot was observed after the addition of biomass to the rice plants.

**Conclusions:**

We conclude that the two stage treatment of dairy effluent is highly effective in removal of BOD and COD besides nutrients like nitrates and phosphates. The treatment also helps in discharging treated waste water safely into the receiving water bodies since it is non toxic for aquatic life. Further, the algal biomass separated after first stage of treatment was highly capable of increasing the growth of rice plants because of nitrogen fixation ability of the green alga and offers a great potential as a biofertilizer.

## Background

Agro-food industries like the dairy and brewery sectors produce effluents with high organic load. These effluents contain high BOD, COD and nutrients such as phosphates, ammonia and/or nitrate, pose a serious threat to the water quality and should be appropriately treated before discharging into the environment [1]. There are several physical, chemical and biological methods for the treatment of effluents derived from the agro-food industries. Biological methods are proved to be much eco friendly when compared with other types of treatments [2,3].

Research suggests that N- and P-rich wastewater is also considered as valuable substrate for cultivation of microalgae which is a type of biological treatment [4]. Microalgae are mostly suspension-type microorganisms and efficient solar driven cell factories that can be useful as treating agents for wastewater. The cultivation of algae in wastewaters offers the combined advantages of treating the wastewaters and simultaneously producing algal biomass, which can further be exploited for protein complements and food additives for aquaculture, animal and human feed, energies such as biogas and fuels and biofertilizer [5]. Little information is available on the use of *Chlorella pyrenoidosa* as a potential agent for treatment of various types of waste waters among the several microalgae used to treat effluents [6].

Separation of algal biomass from the treated water discharge is the key for the success of waste water treatment. Numerous efforts have been devoted to developing a suitable technology for harvesting microalgae. The use of industrial filtration and centrifugation is not cost effective for wastewater treatment. In this context, immobilization of algal cells has been proposed for overcoming the harvest problem as well as retaining the high-value algal biomass for further processing [7].

Among all unicellular algal species, *Chlorella* is a common and effective species for the immobilization and nutrient removal purposes [8]. Uptake rate of phosphorous was low when compared with Nitrogen for *Chlorella sp* from waste waters [9,10]. There are various methods of immobilization out of which calcium alginate is a very simple and cost-effective method, to entrap microbial cells as alginate beads.

Many green algae are also capable of using atmospheric dinitrogen (N_2_) as the source of nitrogen. This important characteristic of nitrogen fixation has enhanced the agro production when green alga is used in agriculture [11]. Many studies have been reported on the use of dried green alga to inoculate soils as a means of aiding fertility. Algalization is the term applied to the use of a defined mixture of algal species to inoculate soil. A 15-20% increase in rice grain yield was observed because of algalization [12,13].

Reduction of BOD and COD is one of the major challenges in wastewater treatment. If effluent with high BOD is discharged into environment, it will increase the bacterial growth in the water while reducing the dissolved oxygen levels in the water that may prove lethal to aquatic life. Much like BOD, a high COD can also deprive aquatic organisms of oxygen needed for survival [14].

In this study, we made an attempt to treat the diary effluent by a novel, low cost photobioreactor using immobilized *C. pyrenoidosa* and evaluate the potential of dairy effluent as a nutrient medium for microalgae growth. The efficacy of sand bed filtration to reduce/remove some of the organic compounds present in the dairy effluent was assessed. The treated dairy effluent is used to check the survival of zebrafish (*Danio rario*) whose gene sequence is identical with humans to assess the biosafety to aquatic life. In addition, the biomass produced is analyzed for its composition, and amenable to be used as fertilizer.

## Materials and methods

### Microalgae

*Chlorella pyrenoidosa* sp. (NCIM NO: 2738) was obtained from National Centre for Industrial Microorganisms (NCIM), Pune, India. Stock culture of *Chlorella pyrenoidosa* was grown photoautotrophically in BG11 media at 28°C under continuous light illumination in four 100 ml borosil flasks. Each liter of the BG11 medium contained NaNO_3_-1.5 g, K_2_HPO_4_-0.04 g, MgSO_4_•7H_2_O-0.075 g, CaCl_2_•2H2O-0.036 g, Citricacid-0.006 g, NaCO_3_-0.02 g, H_3_BO_3_-0.00286 g, MnCl_2_•4H_2_O-0.00181 g, ZnSO_4_•7H2O-0.00022 g, Na_2_MoO_4_•2H_2_O-0.00039 g, CuSO_4_•5H_2_O-0.00008 g, Co(NO_3_)_2_•6H_2_O-0.00005 g, (NH_4_)_6_Mo_7_O_24_.4H_2_O-0.003 g, Na_2_EDTA-0.00001 g. The inoculum was prepared by transferring the cells from stock culture, and incubated aseptically in a 1000 ml flask containing 700 ml of fresh BG11 media under continuous illumination of 34 μmol m^-2^ s^-1^ at 28°C for four days on an orbital shaker set at 120 rpm. A 4 day old culture was used as inoculum at 10% volume for the preparation of immobilized microalgal beads. Gel entrapment is done using 2% Sodium alginate and 0.1 M Calcium chloride. The average diameter of the algal beads inoculated was 0.556 m. Untreated dairy effluent is collected from Vyshnavi dairy, Khammam, Andhrapradesh, India.

### Fish collection

Specimens of zebra fish (4–5 cm long; weight 1.25 ± 0.25 g) were used for toxicity tests. These were procured from a local aquarium shop, Hyderabad were collected from. The fish were transported from the farm in oxygenated polythene bags to the laboratory and immediately transferred into 20 L capacity containing well –aerated unchlorinated ground water. The fish were allowed to acclimatize for 15 days before the experiments. They were fed with rice bran during the acclimation period. Only healthy and active fishes were used for experiments. The temperature, pH, dissolved oxygen, used for acclimatization were 29°C, 7.2 ± 0.2, 5.0-6.5 mg/L respectively.

### Photobioreactor

**Figure 1:**
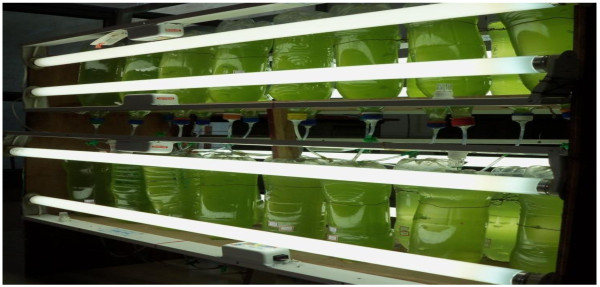
**Photo bioreactors with immobilized microalgal beads.** Inverted polycarbonate bottles on a wooden rack with immobilized microalgal beads for treatment of dairy effluent.

The immobilized beads are weighed approximately to 50 gm and inoculated in 10 one litre inverted polycarbonate bottles set on a wooden rack (Figure 1). A light intensity of 55 μmol m^-2^ s^-1^ was maintained at 28°C for four days. Artificial air supply is provided through air pumps at 1 L min^-1^ for all 10 bottles continuously to ensure the suspension of immobilized beads in dairy water and thorough contact of beads with the nutrients present in dairy effluent. The filtered dairy water is analyzed for its biochemical composition.

### Sand bed filtration

**Figure 2:**
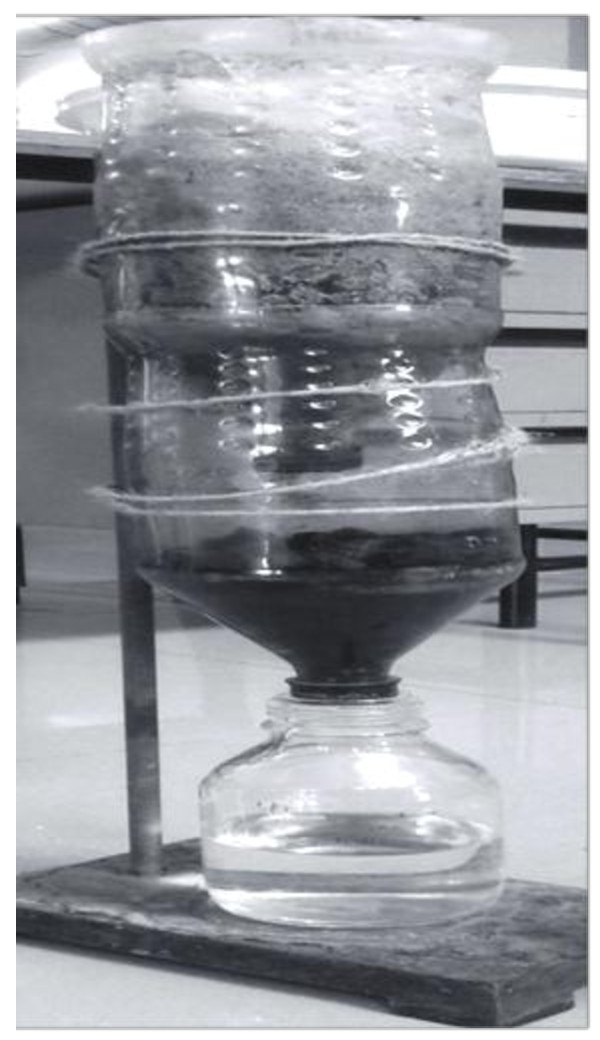
**Low cost two column sand bed filtration.** First column filled with stones and sand and second column spread with charcoal.

A two column sand bed is prepared for the filtration of algal treated dairy water. The first column contains 3 layers of small, medium and large stones each 4 cm and again each layer is covered by fine sand of about 2 cm in height (Figure 2). In the second column of sand bed filtration a charcoal bed is spread. The treated water from the first stage slowly drips into the second stage and the treated water will be collected finally into a borosil bottle after passing through charcoal bed. The treated dairy effluent after sand bed filtration was again analyzed and compared with control.

### Estimation of lipid, carbohydrate and protein

The carbohydrate content was analyzed based on the procedure published by Miao and Wu [15]. In short, 0.1 g dried algal pellet was acidified by adding 20 ml 2.5 M HCl. The acidified solution was then hydrolyzed at 100°C for 30 min and neutralized to pH 7. The volume was adjusted to 100 ml. The filtered sample was subjected to 3,5-dinitrosalicylic acid (DNS) assay [16].

Lipid extraction was performed in a way that was similar to the procedures reported by earlier studies [17,18]. Briefly, 1.0 g dried cells were ground in a mortar and pestle. The dried powder is then transferred to a 50 ml glass centrifuge tube. Chloroform was then added to the tube to make the chloroform/ methanol (2:1, v/v). The tube was vortexed for 5 min and was allowed to stand for 24 h. After that, the tube was centrifuged at 4,000 g for 15 min to remove the algal solids. The supernatant was collected and the solvent was vaporized using Rotovap at 65°C. Oil left in the flask without solvent was weighed to calculate lipid content.

Protein estimation is done using Lowry’s method [19]. Briefly, 0.1 g algal powder is taken and 2 ml of 5% TCA is added. The mixture is centrifuged at 2000 rpm for 10 min. Solution containing pellet is dissolved in 1.5 ml of 0.1 N NaOH. From the above prepared solution 0.2 ml is taken into 20 ml test tube and 5 ml of alkaline copper solution is added. After incubation at room temperature for 15 min, 0.5 ml of Folin-Ciocalten reagent is added. Again the mixture is incubated for 30 min and OD values were taken at 540 nm.

## Results and discussion

### Biochemical analysis of algal biomass

**Figure 3:**
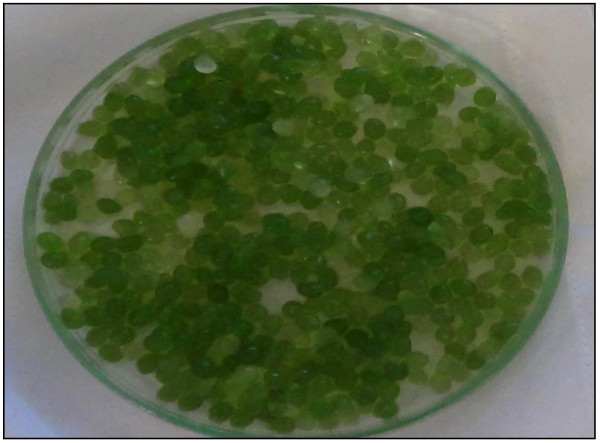
**Wet algal beads separated after four days of cultivation.** The fully bulged algal beads of diameter 1.078 m were filtered after four days of cultivation in dairy effluent. The initial bead size before inoculation into photo bioreactor was 0.556 m.

After four days of the growth period the algal beads were separated from the dairy water effluent using nylon filter cloth (Figure 3). Earlier studies mentioned that even at high concentrations (up to 900 mg/L) of phosphates, immobilized beads tend to be stable in acidic pH (5.5-6.5) ranges up to 4 days [20]. Hence the bead stability was maintained throughout the experiment without cell leakage. The fully bulged algal beads of diameter 1.078 m were analyzed for lipid, carbohydrate and protein content (Table 1). The high protein content of algal biomass was supported by Rodulfo in his earlier studies [21]. The treated water is compared with the control (untreated dairy effluent) after each stage treatment process. A clear liquid without turbidity can be observed after two stage treatment process of dairy effluent.

**Table 1:** Algal biomass analysis

**S.No**	**Biochemical parameter**	**Percentage**
1	Lipid content	10.5
2	Carbohydrate	28
3	Protein	44

The results were an average of three experiments conducted.

### Dairy water analysis

#### Nitrogen and phosphate removal

The phosphates are reduced by 97% and 99% after algal bead treatment and sand bed filtration respectively (Table 2). The NH_4_^+^-N was almost completely reduced after two stage filtration process. There was a significant decrease of N and P which may be because of algal uptake and adsorption on alginate gels were found to be the major processes involved in the removal of N and phosphate in the present study. Previous studies have also reported that air-stripping of ammonia is a possible mechanism for N removal in an intensively aerated microalgal system with alkaline pH resulting from photosynthetic activity and aeration [22-24].

**Table 2:** Water analysis of three different samples

**S.No**	**Parameter**	**Control (untreated dairy effluent)**	**Dairy effluent after algal bead treatment**	**Dairy effluent after sand bed filtration**	**Acceptable limits as per APPCB**
1	BOD(3 @ 27°C)	1200 mg/L	146 mg/L	32 mg/L	<100 mg/L
2	COD	2900 mg/L	448 mg/L	106 mg/L	<250 mg/L
3	NH_4_^+^-N	35 mg/L	0.6 mg/L	0.54 mg/L	<50 mg/L
4	Phosphates	30 mg/L	0.87 mg/L	0.39 mg/L	--
5	pH	6.5	8.01	8.52	5.5-9.0

Sampling and analysis were done as per standard methods prescribed by BIS.

#### Effect on pH

The untreated dairy effluent has an acidic pH value initially but after algal beads treatment and sand bed filtration the treated effluent is shifted to alkaline pH range. NH_4_^+^-N could be lost via ammonia volatilization while PO_4_^3-^-P was removed by chemical precipitation, because of which alkaline pH was recorded in the two stage treatment system.

#### BOD and COD removal

Waste water of dairy industry contains large quantities of milk constituents such as casein, lactose, fat, inorganic salts. All these components contribute largely towards their high biochemical oxygen demand. High BOD and COD values lead to the deprival of oxygen for aquatic life in water [25]. BOD and COD have decreased up to 88% and 85% respectively after algal bead treatment (Table 2). The same were further reduced by 98% and 96% after sand bed filtration which was in acceptable levels prescribed by Andhra Pradesh Pollution Control Board (APPCB), Andhra Pradesh, India. Previous studies also showed that algal uptake had little effect on the removal of COD when compared with that of BOD values which is in agreement with our present study [26,27]. Dairy water after algal beads treatment and sand bed filtration each one liter, were used for performing the mortality studies on twenty *Danio rario* (zebra fish). The results were analyzed in comparison with untreated dairy effluent. It was observed that the mortality was 10% after first stage of treatment and zebra fish survival percentage increased to 100% (Table 3) after second stage treatment which proved that the two stage treated water is non toxic to aquatic life.

**Table 3:** **Relative toxic potential of dairy effluent to *Danio rario***

**S.No**	**Sample**	**Survival percent**				**Toxic potential**
		**24 h**	**48 h**	**72 h**	**96 h**	
1	Untreated Dairy effluent	0	0	0	0	Highly Toxic
2	Dairy effluent after algal bead treatment	100	90	90	90	Acceptable
3	Dairy effluent after Sand bed filtration	100	100	100	100	Non-toxic

### Algal biomass as biofertilizer

The algal biomass is also tested for its effectiveness as biofertiliser. A control is taken without adding biofertiliser to the rice seedlings. Another sample is taken with the same number of rice seedlings and 20 gms of dried algal beads were added as biofertilizer. After 10 days of growth the average length of root and shoot were measured and recorded (Table 4).

**Table 4:** Lengths of root and shoot in rice plant

**S.No**	**Parameter**	**Control (without biofertilizer)**	**Test (with biofertilizer)**
1	Root	2 cm	4 cm
2	Shoot	10.5 cm	15 cm

Green alga can photosynthesize and fix nitrogen, and these abilities, together with great adaptability to various soil types, make them ubiquitous. Ammonia can be taken up by green alga through passive diffusion or as ammonium (NH_4_^+^) by a specific uptake system [11].

## Conclusion

The present study demonstrated that two stage treatment was very effective in removing NH_4_^+^-N and phosphate from wastewater. A complete removal of NH_4_^+^-N (100%) and 99% reduction of PO_4_^3-^-P was achieved within 96 h of treatment. The completely treated dairy effluent was proved to be non toxic for the zebra fish because of the drastic decrease in BOD and COD values (98% and 96% respectively). The algal biomass was higher in protein content (44%) and when the same was used as biofertiliser for rice seeds a 35% increase in growth of the rice plant was observed. Hence the process proved to be useful in treating wastewater for aquatic life and as a biofertiliser.

## Competing interests

The authors declare that they have no competing interests.

## Authors’ contributions

YR has contributed for the concept development, design of the study, conduct of experimental work and drafted the manuscript. HG was involved in conducting a part of experimental work. Both authors read and approved the final manuscript.

## Authors’ information

YR has done her Masters in Chemical engineering currently holds the position of Associate Professor in Biotechnology and pursuing her Ph.D in the area of algal biofuels. HVNG has completed Graduation in Biotechnology and is working along with YR for his Postgraduate Project work.
